# Opportunities and Challenges of Cardiovascular Disease Risk Prediction for Primary Prevention Using Machine Learning and Electronic Health Records: A Systematic Review

**DOI:** 10.31083/RCM37443

**Published:** 2025-04-25

**Authors:** Tianyi Liu, Andrew J. Krentz, Zhiqiang Huo, Vasa Ćurčin

**Affiliations:** ^1^School of Life Course & Population Sciences, King’s College London, SE1 1UL London, UK; ^2^Metadvice, 1025 St-Sulpice, Switzerland

**Keywords:** cardiovascular disease, machine learning, electronic health records, risk prediction, primary prevention

## Abstract

**Background:**

Cardiovascular disease (CVD) remains the foremost cause of morbidity and mortality worldwide. Recent advancements in machine learning (ML) have demonstrated substantial potential in augmenting risk stratification for primary prevention, surpassing conventional statistical models in predictive performance. Thus, integrating ML with Electronic Health Records (EHRs) enables refined risk estimation by leveraging the granularity and breadth of longitudinal individual patient data. However, fundamental barriers persist, including limited generalizability, challenges in interpretability, and the absence of rigorous external validation, all of which impede widespread clinical deployment.

**Methods:**

This review adheres to the methodological rigor of the Preferred Reporting Items for Systematic Reviews and Meta-Analyses (PRISMA) and Scale for the Assessment of Narrative Review Articles (SANRA) guidelines. A systematic literature search was performed in March 2024, encompassing the Medline and Embase databases, to identify studies published since 2010. Supplementary references were retrieved from the Institute for Scientific Information (ISI) Web of Science, and manual searches were curated. The selection process, conducted via Rayyan, focused on systematic and narrative reviews evaluating ML-driven models for long-term CVD risk prediction within primary prevention contexts utilizing EHR data. Studies investigating short-term prognostication, highly specific comorbid cohorts, or conventional models devoid of ML components were excluded.

**Results:**

Following an exhaustive screening of 1757 records, 22 studies met the inclusion criteria. Of these, 10 were systematic reviews (four incorporating meta-analyses), while 12 constituted narrative reviews, with the majority published post-2020. The synthesis underscores the superiority of ML in modeling intricate EHR-derived risk factors, facilitating precision-driven cardiovascular risk assessment. Nonetheless, salient challenges endure heterogeneity in CVD outcome definitions, undermine comparability, data incompleteness and inconsistency compromise model robustness, and a dearth of external validation constrains clinical translatability. Moreover, ethical and regulatory considerations, including algorithmic opacity, equity in predictive performance, and the absence of standardized evaluation frameworks, pose formidable obstacles to seamless integration into clinical workflows.

**Conclusions:**

Despite the transformative potential of ML-based CVD risk prediction, it remains encumbered by methodological, technical, and regulatory impediments that hinder its full-scale adoption into real-world healthcare settings. This review underscores the imperative circumstances for standardized validation protocols, stringent regulatory oversight, and interdisciplinary collaboration to bridge the translational divide. Our findings established an integrative framework for developing, validating, and applying ML-based CVD risk prediction algorithms, addressing both clinical and technical dimensions. To further advance this field, we propose a standardized, transparent, and regulated EHR platform that facilitates fair model evaluation, reproducibility, and clinical translation by providing a high-quality, representative dataset with structured governance and benchmarking mechanisms. Meanwhile, future endeavors must prioritize enhancing model transparency, mitigating biases, and ensuring adaptability to heterogeneous clinical populations, fostering equitable and evidence-based implementation of ML-driven predictive analytics in cardiovascular medicine.

## 1. Introduction

Cardiovascular disease (CVD) remains the most significant threat to global population health and has seen an emergent increase in negative impact [1]. One strategy might involve early prediction of CVD risk and prevention before the symptoms of CVD manifest, through prescribing statins and lifestyle intervention [2, 3, 4].

In most Western countries, clinical guidelines have discussed and suggested that primary care utilize CVD risk prediction scores, usually focusing on the individual’s 10-year future risk of CVD based on basic indices such as blood pressure and their previous medical history. For instance, QRISK3 [5] in the UK by NICE (The National Institute for Health and Care Excellence) guidelines [2], the Pooled Cohort Equations (PCE) in the US by ACC/AHA (American College of Cardiology/American Heart Association) guidelines [3], and SCORE [6] in Europe by ESC (European Society of Cardiology) guidelines [4]. These risk scores are all based on conventional statistical models, such as Cox proportional hazards models. These scores have been independently externally validated by various research [7, 8] and have been amended by adding new predictors by developers [9]. Additionally, these scores have been used in clinical settings for years.

However, several studies have shown that the performance of these scores is not satisfactory, including the underestimation or overestimation of risk for certain population groups [10, 11]. Recent findings suggest that machine learning (ML) might be a good method to replace conventional statistical algorithms due to its ability to handle more complex data types [12]. And electronic health records (EHRs) might be a great source for this new technique to achieve this task [13].

### 1.1 Rationale

The motivation and rationale for this review are grounded in the imperative to enhance the performance of CVD risk prediction. In this context, “performance” encompasses not only the statistical metrics of discrimination and calibration but also the practical applicability and effectiveness of algorithms in real-world clinical settings. The increasing prevalence of CVD underscores the urgency to explore innovative approaches to predicting and managing CVD. The adoption of EHRs and ML technologies has shown promise in refining CVD risk prediction. Despite their potential advantages, the use of these technologies is not without challenges and limitations.

Thus, this review seeks to offer a comprehensive examination of both the potential benefits and the limitations associated with employing EHRs and ML for CVD risk prediction, while also identifying opportunities and challenges for future research endeavours. By enlightening healthcare professionals and researchers about the capabilities of these technologies, we aim to enhance their utilization and, ultimately, improve patient outcomes through improved risk prediction and management strategies.

### 1.2 Objectives

The objectives of the review are to:

- 1. Examine the current evidence on CVD prediction models and assess the potential of EHRs and ML models for enhancing CVD risk prediction.
- 2. Identify the limitations of using EHRs and ML for CVD risk prediction, covering both clinical and technical aspects.
- 3. Identify elements of an integrative framework for development, validation, and application of ML based CVD risk prediction algorithm.
- 4. Highlight areas for future research directions to optimize the use of EHRs and ML for CVD risk prediction.

## 2. Methods

This review is conducted based upon the elements which described in the Preferred Reporting Items for Systematic Reviews and Meta-Analyses (PRISMA) [14] and Scale for the Assessment of Narrative Review Articles (SANRA) [15].

### 2.1 Study Design and Search Strategy

We conducted our research on publications in March 2024. Firstly, we searched electronic databases, including Medline and Embase via Ovid, for the period from January 2010 to the present. We utilized a combination of Medical Subject Headings (MeSH), and free text related to ‘CVD’, ‘ML’, ‘EHR’, and ‘risk assessment/factors’ to identify relevant studies published since 2010. The search was limited to the selected years, as more contemporary studies are likely to utilize ML algorithms and EHR datasets.

We further refined our research by focusing on publications that involved human subjects, were written in English, and had full-text availability. We extracted the necessary abstract information from these publications. Details of the search log and strategies are available in the Appendix A. Additionally, we conducted a comprehensive search of the reference lists from the selected papers using Institute for Scientific Information (ISI) Web of Science. Based on our knowledge of the research topic, we manually identified and included potential publications of interest.

### 2.2 Criteria for Study Selection 

We extracted all the search results and materials collected from multiple sources and removed any duplicate papers using Rayyan [16], an online application for the initial screening of systematic reviews. Firstly, we screened the titles and abstracts, eliminating any irrelevant publications. Subsequently, we reviewed the full text to extract potentially inclusive studies, enabling us to select the best source materials for inclusion.

The primary interest focuses on ML-based prediction models utilizing dataset derived from EHR, specifically targeting algorithms for the primary prevention of CVD by predicting the long-term incidence of major cardiovascular events. Thus, eligible citations should include qualitative reviews discussing this area or systematic reviews, with or without quantitative meta-analysis, reporting on such models. Studies that solely develop or validate individual models, as well as methodology papers, will not be considered. The target population should be adults with no prior CVD history or any cardiovascular (CV) symptoms (e.g., acute coronary syndrome (ACS)) or those on statin prescriptions. Models developed exclusively for patients with specific comorbidities (e.g., diabetes mellitus (DM), chronic kidney disease (CKD)) or for certain sub-population groups (e.g., minor ethnic groups, gender-specific) will not be included. The models must be suitable for use in predicting long-term outcomes (e.g., 5, 10, 15 years risk, lifetime risk) in outpatient/general practice (GP) settings for the purpose of early prevention, rather than for patients in inpatient hospitalization or emergency departments (ED), where the aim is to predict short-term adverse health outcomes. The reviews or systematic reviews discussed must involve ML/deep learning (DL), with at least a portion of the models discussed being ML-based. Studies reporting only conventional statistical methods, such as survival analysis and cox model, will not be included. Likewise, studies focusing solely on artificial intelligence (AI) in the context of embedding, natural language processing (NLP) subtype definition clustering, will be excluded. The CVD outcomes of interest should include either composite or individual cases of coronary heart disease (CHD), stroke/transient ischemic attack (TIA) and heart failure, excluding management missions such as predicting length of stay, admission or readmission, and mortality or survival after medical operations in hospitalization and ED settings. The required data type should be based on EHR. While this is a novel technique and most data sources will be structured patient-level health data, the review or systematic review must at least mention and discuss EHR data. Studies that do not mention EHR will be excluded. Data sources focusing only on images, sound, and genetic data will not be considered unless combined with EHR data. Note, these inclusion criteria are not overly strict; studies that discuss or mention ML, CVD risk, and EHR in some capacity will be considered. Detailed inclusion and exclusion criteria are presented in Table 1.

**Table 1.** **Inclusion and exclusion criteria**.

	Inclusion criteria	Exclusion criteria
Publication type	Review, systematic review with or without meta-analysis.	Individual model development, validation, methodology studies, editorial comments, and research protocols.
Populations	Adult (18 years of age and older), asymptomatic.	Patients with prior CVD, CV symptoms, or those on statin prescriptions.
Settings	Outpatients/GP.	Inpatients/hospitalizations, ED, or remote monitoring at home.
Models and tasks	Multivariable ML/DL models for long-term individual risk prediction.	Studies that only report conventional statistical methods, feature selection for risk prediction, and ML models for embedding, NLP, and subtype definition/clustering.
CVD outcomes	CHD, stroke/TIA, or heart failure.	In-hospital CVD outcomes including survival/mortality after surgery, (re)admission, and length of stay.
Data type	Electronic health (medical) records.	Studies incorporating only ECG, echocardiograms, ultrasounds, sound, DNA sequences, and image data.
		Studies that discuss registries but do not mention EHR at all.
Filter applied	Published after January 2010, publication in English, human studies, full-text available, peer-reviewed literature.	

CVD, cardiovascular disease; GP, general practice; ML, machine learning; DL, deep learning; ED, emergency departments; EHR, Electronic Health Record; CV, cardiovascular; NLP, natural language processing; TIA, transient ischemic attack; CHD, coronary heart disease; ECG, electrocardiogram.

### 2.3 Data Extraction

We utilized the PRISMA guidelines [14] and Microsoft Excel to extract data from the included publications. From each paper, we recorded pertinent information such as the first author’s name, year of publication, and the journal in which the study was published. We also noted whether each publication contained key details related to the domains we were particularly interested in. Additionally, we considered the rationale and objectives outlined in the introductions of the papers. Furthermore, we extracted the main findings of each study and provided a summary.

## 3. Results

### 3.1 Study Selection

**Fig. 1.**
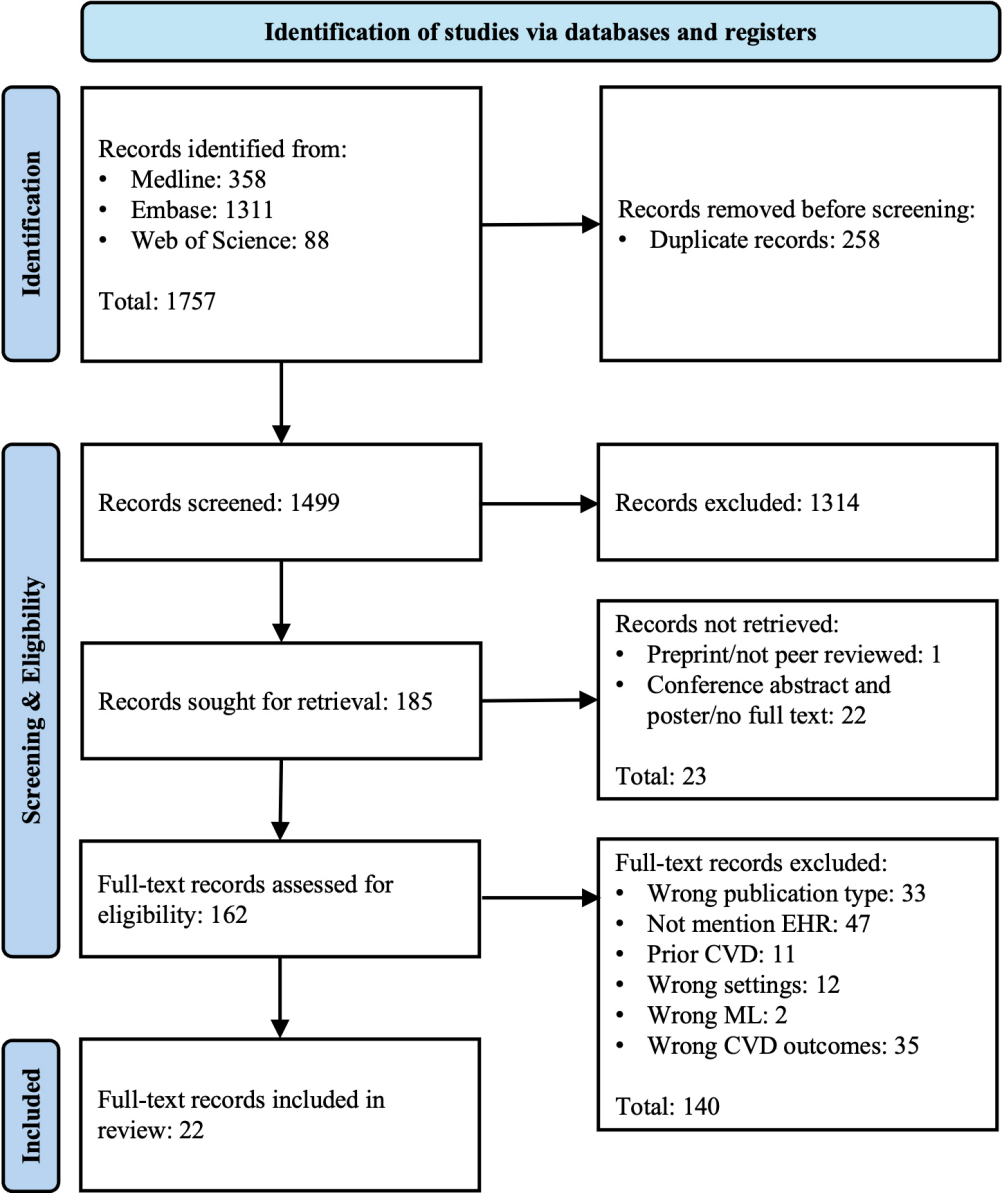
**PRISMA flow diagram**. PRISMA, Preferred Reporting Items for Systematic Reviews and Meta-Analyses.

The study selection process is depicted in the Fig. 1. A total of 1757 studies were identified from all considered sources: 358 from PubMed/Medline, 1311 from Embase, and an additional 88 citations through backward searching in the Web of Science. After the removal of 258 duplicate studies, 1499 underwent an in-depth evaluation based on title and abstract screening. During this phase, 1314 publications were excluded. Subsequently, 185 records were sought for retrieval; however, 23 additional studies were excluded because they were either preprints without peer review or were conference abstracts and posters. As a result, 162 studies were deemed eligible for further full-text assessment of their eligibility. Ultimately, after applying all exclusion criteria, 140 publications were excluded. The Table 2 (Ref. [11, 17, 18, 19, 20, 21, 22, 23, 24, 25, 26, 27, 28, 29, 30, 31, 32, 33, 34, 35, 36, 37]) provides a summary of the characteristics of the remaining 22 publications [11, 17, 18, 19, 20, 21, 22, 23, 24, 25, 26, 27, 28, 29, 30, 31, 32, 33, 34, 35, 36, 37].

**Table 2.** **Summary of characteristics of publications included in the analysis**.

Reference			Concept			Infor extraction
Title (Author year)	Journal	Review type	CVD outcomes	Machine learning	EHR relevant	Field of interest and main conclusion
Social determinants in machine learning cardiovascular disease prediction models: a systematic review	American Journal of Preventive Determinants Medicine	Systematic review	All CVD	All ML	Mentioned	Most studies included in this review showed that, when comparing multiple models within the study, ML models, especially NNs, demonstrated superior performance compared to conventional models.
(Yuan Zhao *et al*. 2021 [20])						SDH were predominantly found to improve model performance. ML may be more aptly suited to encapsulate the complex nature of SDH due to its inherent flexibility.
						EHR should be recognized as a potential source for a diverse range of SDH. Furthermore, neighbourhood/community-based SDH should be explored beyond individual-level SDH, such as gender and ethnicity, which are currently the most commonly utilized predictors in existing prediction models.
Artificial intelligence and prediction of cardiometabolic disease: systematic review of model performance and potential benefits in indigenous populations	Artificial Intelligence in Medicine	Systematic review	All CVD	All ML	Mentioned	Compared to conventional models, ML risk prediction models are capable of indiscriminately processing all data from EHRs and demonstrate superior performance within the selected studies. However, most selected studies have inadequately reported on missing data, or the methods used to address it.
(Keunwoo Jeong *et al*. 2023 [21])						The AUC was the most commonly used metric to measure model performance, yet it does not provide clinical value without further interpretation, and establishing a threshold proves challenging.
						This review also discuss that AI models may fail to accurately represent minority ethnicities (Indigenous) due to insufficient EHR data, lack of external validation in these populations. This issue could potentially be resolved by linking EHRs to regional primary care systems.
A systematic review on machine learning approaches for cardiovascular disease prediction using medical big data	Medical Engineering and Physics	Systematic review	All CVD	All ML	Mentioned	Machine learning improves CVD prediction accuracy by leveraging “experience” derived from EHR.
(Javed Azmi *et al*. 2022 [22])						Gaps identified in selected literatures include considerations of smaller data size, imbalance in data after splitting, inconsistent model performance, and statistical heterogeneity caused by hyperparameter tuning.
						It also underscores the importance of utilizing feature selection and ensemble techniques. Additionally, a challenge arises when findings from selected literature do not align with current clinical practice, and reports tend to focus solely on technical components without incorporating clinical features, attributed to a lack of clinical monitoring.
Machine learning for subtype definition and risk prediction in heart failure, acute coronary syndromes and atrial fibrillation: systematic review of validity and clinical utility	BMC Medicine	Systematic review	All CVD	All ML	Discussed	Based on the comprehensive ML-specific checklist developed by the authors, selected risk prediction studies exhibited limitations in development, validation, and impact, which are crucial for the algorithm’s clinical utility and implementation.
(Amitava Banerjee *et al*. 2021 [23])						EHR data have been either underutilized or underreported in the included studies. However, its diversity in data and settings can offer ML the opportunity to predict various co-existing types of CVD and externally validate across countries.
						The majority of studies overestimate potential healthcare impact and lack translation from science to practice, revealing a gap in generalizability, applicability, and clinical utility. Guidelines may address this issue.
Machine learning prediction in cardiovascular diseases: a meta-analysis	Scientific Reports	Systematic review	All CVD	All ML	Mentioned	The predictive performance of ML in CVD shows promise, particularly with SVM and boosting algorithms in terms of AUC. However, selecting an optimal AUC threshold and interpreting it remains ambiguous in clinical practice.
(Chayakrit Krittanawong *et al*. 2020 [24])		Meta-analysis				Heterogeneity among ML models arises from the lack of disclosure of hyperparameters and feature selection in many selected studies. Moreover, some reports emphasize only technical features, omitting clinical features.
						Integrating ML with EHRs will be clinically practical, as EHR is readily accessible and contains a large number of predictors.
Effectiveness of artificial intelligence models for cardiovascular disease prediction: network meta-analysis	Computational Intelligence and Neuroscience	Systematic review	All CVD	All ML	Mentioned	DL models have shown more promising results compared to traditional ML models, particularly in terms of AUC. However, the synthesis of selected studies indicates that for predicting different types of CVD, the optimal ML model varies.
(Yahia Baashar *et al*. 2022 [28])		Meta-analysis				By integrating clinical data and physicians’ reports from EHR, ML, especially DL, is recognized as a promising method for enhancing the accuracy of various CVD tasks and facilitating its application in clinical settings.
Prediction models for heart failure in the community: a systematic review and meta-analysis	European Journal of Heart Failure	Systematic review	Heart failure	All ML	Reference	ML applied to gender, ethnicity, and CVD phenotyping has demonstrated excellent predictive performance for incident HF, highlighting the shift towards data-driven computational modelling for prediction in CVD.
(Ramesh Nadarajah *et al*. 2023 [30])		Meta-analysis		Conventional		Over half of these studies utilize data derived from EHR. However, the high risk of bias, missingness of requisite variables in routinely collected data, low certainty of evidence, and a lack of impact studies mean that the utility of integrating HF prediction models into clinical practice remains uncertain.
Machine-learning versus traditional approaches for atherosclerotic cardiovascular risk prognostication in primary prevention cohorts: a systematic review and meta-analysis	European Heart Journal - Quality of Care and Clinical Outcomes	Systematic review	Risk	All ML	Reference	The enhanced accuracy of ML algorithms, along with their capacity to utilize EHRs as data-rich environments, indicates their potential as the future of ASCVD risk prediction. Studies included in the analysis have consistently demonstrated superior calibration when compared to traditional risk scores.
(Weber Liu *et al*. 2023 [32])		Meta-analysis		Conventional		However, challenges persist among the included models, such as inconsistent reporting of performance metrics, limited access to datasets, a high risk of bias, and inadequate disclosure of the models’ technical details. These issues hinder the models’ explainability, replicability, transparency, and clinical implementation.
Artificial intelligence in the risk prediction models of cardiovascular disease and development of an independent validation screening tool: a systematic review	BMC Medicine	Systematic review	All CVD	All ML	Reference	AI/ML has become a promising tool in CVD risk prediction, with most studies included leveraging EHR data. However, the selected models exhibit geographical imbalances and a significant lack of external validation, transparency, interpretability, and reproducibility.
(Yue Cai *et al*. 2024 [35])						Furthermore, there is a noticeable absence of independent external validation for existing models. This trend suggests that researchers in the field of AI risk prediction may prioritize developing new models over validating existing ones. Nonetheless, validation is crucial for informing clinical decisions. Despite its potential, this field is still in its nascent stages.
Applications of artificial intelligence/machine learning approaches in cardiovascular medicine: a systematic review with recommendations	European Heart Journal - Digital Health	Systematic review	All CVD	All ML	Reference	The superiority of AI/ML methods, especially when integrating EHR, is often proclaimed in comparison to conventional methods. However, this claimed superiority is frequently based on subjective definitions provided by the authors. Proper evaluation of an ML algorithm, both individually and in comparison, with alternative algorithms, requires metrics that comprehensively address calibration and discrimination. This necessitates detailed reporting of evaluations to ensure data and methodological transparency, thereby promoting reproducibility. Additionally, the evaluation and application of these methods should adhere to established clinical guidelines. Yet, these standards have not been consistently met, leading to the underutilization of these advanced methods in clinical practice.
(Sarah Friedrich *et al*. 2021 [36])						
Prioritizing the primary prevention of heart failure: measuring, modifying and monitoring risk	Progress in Cardiovascular Diseases	Narrative review	All CVD	All ML	Mentioned	Focus should shift to primary prevention of HF, targeting individuals at risk in stages A and B. Currently, a CVD risk score has not yet been adopted for HF in clinical practice. The heterogeneity of HF, the lack of standardized definitions, and the limited datasets with adjudicated HF outcome data have made developing and implementing an accurate risk prediction model challenging. Much research now utilizes EHR and ML to accurately predict long-term HF outcomes.
(Ruchi Patel *et al*. 2024 [18])				Conventional		
Understanding the bias in machine learning systems for cardiovascular disease risk assessment: the first of its kind review	Computers in Biology and Medicine	Narrative review	All CVD	All ML	Reference	Conventional risk scores are unable to handle non-linear relationships and lack granularity in assessing CVD risk. This ML system designs models using CVD risk factors to predict cardiovascular risks. Key factors influencing these models include outcome definitions, classifier types, prediction timeframes, validation methods, and clinical evaluations. Popular algorithms such as XGBoost, SVM, RF, and NN are widely used. With the advent of AI, there has been a shift towards applying these systems in clinical settings. However, this often overemphasizes accuracy while underemphasizing the validation of AI systems, leading to biases in AI applications.
(Jasjit S. Suri *et al*. 2022 [19])						
A review of risk prediction models in cardiovascular disease: conventional approach vs. artiﬁcial intelligent approach	Computer Methods and Programs in Biomedicine	Narrative review	All CVD	All ML	Mentioned	The optimal performance of a prediction model hinges on several factors: the model’s objectives, its ability to generalize and remain robust across different scenarios, and its reproducibility in real-world clinical settings. AUC is an effective metric for evaluating ML models in CVD. Common risk factors considered in these models include age, sex, and ethnicity. Adherence to data sharing guidelines is essential for acquiring and standardizing data, which supports the ML process while ensuring data security and privacy. Although DL offers improved performance over traditional machine learning, it requires more extensive data for training. The application of ML for CVD is less prevalent in developing countries, where resources and data availability may be limited. Looking forward, the integration of these models with EHR could enhance future prospects and utility.
(Aizatual Shafiqah Mohd Faizal *et al*. 2021 [17])						
Machine learning and the conundrum of stroke risk prediction	Arrhythmia & Electrophysiology Review	Narrative review	Stroke	All ML	Mentioned	ML in healthcare, particularly in CVD risk assessment, leverages complex data from EHRs to model relationships and predict outcomes. While traditional CVD models use limited predictors and may oversimplify, ML algorithms can handle multiple, intricate risk factors and improve prediction accuracy. These ML models are powerful tools for identifying patients at risk and enabling early interventions, potentially reducing healthcare system strain. However, their “black box” nature makes them difficult to interpret, and further research is needed to validate their efficacy across diverse populations and enhance their transparency before they can be fully integrated into clinical practice.
(Yaacoub Chahine *et al*. 2023 [25])						
Artificial intelligence-based clinical decision support systems in cardiovascular diseases	The Anatolian Journal of Cardiology	Narrative review	All CVD	All ML	Mentioned	AI systems integrated with EHRs are enhancing CVD risk assessment by leveraging patient data like age, sex, medical history, and lifestyle. These systems provide personalized, evidence-based recommendations for CVD prevention, streamlining clinicians’ workflows and promoting early interventions. The integration of Clinical Decision Support Systems (CDSS) into EHRs facilitates the adoption of healthy lifestyle practices, offering specific guidance on diet and exercise. However, the use of such AI systems requires stringent data security and updated training for healthcare professionals to ensure effective and responsible use.
(Serdar Bozyel *et al*. 2024 [26])						
Artificial intelligence in cardiovascular prevention: new ways will open new doors	Journal of Cardiovascular Medicine	Narrative review	All CVD	All ML	Mentioned	Artificial intelligence is revolutionizing cardiovascular prevention, enhancing patient healthcare, and improving cost-effectiveness. It allows for rapid analysis of extensive medical histories and integrates this data with EHR systems to provide clinicians with crucial insights. Moreover, combining machine learning with clinical risk scoring can reduce the number needed for screening and boost the effectiveness of AF screening. However, adopting AI for decision-making faces challenges such as when to apply these techniques, how to interpret results, and translating them into clinical practice. Data protection, transparency, responsibility, and trustworthiness require attention due to the lack of standardization and interoperability across institutions.
(Michele Ciccarelli *et al*. 2023 [27])						
New strategies and therapies for the prevention of heart failure in high‐risk patients	Clinical Cardiology	Narrative review	Heart failure	All ML	Mentioned	Focusing on primary prevention of HF in early stages (A and B) can significantly delay progression. Current HF prevention lacks the risk-based approach widely accepted in atherosclerotic cardiovascular disease prevention. Machine learning ML models, leveraging data from EHRs, offer the potential for real-time, dynamic risk assessment. They enable continuous updates as new data emerge, providing a robust tool for long-term risk prediction, crucial given the lifetime risk of HF. Integrating ML models into EHRs will improve data capture and risk estimation, helping to identify individuals at both immediate and long-term risk, thus enhancing targeted prevention efforts.
(Michael M. Hammond *et al*. 2022 [29])				Conventional		
Artificial intelligence and heart failure: a state-of-the-art review	European Journal of Heart Failure	Narrative review	Heart failure	All ML	Mentioned	The integration of AI in HF management, particularly for early stages A and B, could significantly enhance diagnosis and treatment. AI algorithms excel in managing large datasets from EHRs, allowing for advanced risk stratification and timely interventions. These capabilities not only help in tailoring individualized treatment plans but also in optimizing healthcare resources. However, AI models face challenges such as ensuring interpretability, requiring rigorous validation to prove reliability and effectiveness in diverse clinical settings. Regulatory oversight is critical, emphasizing the need for privacy, security, and ethical considerations in algorithm training. Overall, AI promises to transform HF care, necessitating collaboration between clinicians and data scientists to ensure its effective clinical integration.
(Muhammad Shahzeb Khan *et al*. 2023 [37])						
Use of multi-modal data and machine learning to improve cardiovascular disease care	Frontiers in Cardiovascular Medicine	Narrative review	All CVD	All ML	Mentioned	The integration of ML in CVD management through EHRs is transforming risk assessment and treatment. By leveraging diverse data sources, including imaging and genetic data, ML models can predict long-term risks like ischemic heart disease more accurately than traditional methods. These advanced models, developed from fused data from EHRs and other modalities like CT scans, not only enhance the understanding of risk factors but also allow for real-time, personalized healthcare interventions. However, these systems require careful handling of data quality and ethical considerations to ensure they are effective and fair. As technology progresses, the potential for ML in improving cardiovascular healthcare by providing more accurate and timely diagnoses continues to grow.
(Saeed Amal *et al*. 2022 [31])						
Reviewing the use and quality of machine learning in developing clinical prediction models for cardiovascular disease	Postgraduate Medical Journal	Narrative review	Risk	All ML	Mentioned	ML offers a powerful upgrade to conventional CVD risk assessments like the ACC/AHA equations by analysing complex data from EHRs. While ML can handle more variables and complex interactions, it faces challenges like potential overfitting and the opaque nature of some algorithms. For effective clinical adoption, ML models must be validated against large, diverse datasets and conform to transparency standards like TRIPOD. This validation and adherence to high-quality reporting will help integrate ML into mainstream medicine, enhancing predictive accuracy and patient care.
(Simon Allan *et al*. 2022 [11])						
Polysocial risk scores: implications for cardiovascular disease risk assessment and management	Current Atherosclerosis Reports	Narrative review	All CVD	All ML	Discussed	The integration of ML and EHR is revolutionizing CVD risk assessment by developing polysocial risk scores (pSRS). These scores leverage a wide array of data from EHRs, including social determinants of health (SDOH), to enhance prediction and management of CVD. ML algorithms process vast datasets to identify critical SDOH predictors, improving the personalization of healthcare interventions. This novel approach supports more holistic care models that address both clinical and social risk factors, aiming to improve patient outcomes significantly. With continuous updates and learning from real-world data, ML-enhanced pSRS could lead to more equitable and effective cardiovascular health management.
(Zulqarnain Javed *et al*. 2023 [33])				Conventional		
Machine learning in cardiovascular risk prediction and precision preventive approaches	Current Atherosclerosis Reports	Narrative review	All CVD	All ML	Mentioned	ML is significantly advancing CVD risk assessment by integrating a broad range of data from EHRs. ML models excel in analysing complex, multifaceted data including demographic, clinical, and social determinants of health, enhancing traditional risk prediction methods like the Framingham risk score. These models support the development of personalized risk profiles, improving prediction accuracy and treatment strategies. As ML continues to evolve, it promises to refine how we understand and manage CVD, making healthcare more personalized and effective by utilizing comprehensive data analysis. This approach not only promises better patient outcomes but also aids in the shift towards more holistic cardiovascular health management.
(Nitesh Gautam *et al*. 2023 [34])						

HF, heart failure; ACC/AHA, American College of Cardiology/American Heart Association; NN; neural network; AUC, area under the receiver operating characteristic curve; SDH, social determinants of health; XGBoost, extreme gradient boosting; SVM, support vector machine; RF, random forest; CT, clinical trial; TRIPOD, Transparent Reporting of a multivariable prediction model for Individual Prognosis Or Diagnosis; ASCVD, atherosclerotic cardiovascular disease; AI, artificial intelligence.

### 3.2 Descriptive Results

Out of the 22 selected publications, 10 (45%) are systematic reviews. Among these, 4 also include quantitative synthesis via meta-analysis and 5 of them register with PROSPERRO [38]. The remaining 12 (55%) are categorized as review articles. The majority of these publications are recent, mostly post-2020.

Among the 10 systematic reviews (SRs), most searched for studies within databases such as Medline/PubMed, Embase, and Web of Science (WoS). Only one SR did not adhere to reporting guidelines [22], while the others followed PRISMA [14] or TRIPOD (Transparent Reporting of a multivariable prediction model for Individual Prognosis Or Diagnosis) [39]. For the risk of bias assessment, three used QUADAS (Quality Assessment of Diagnostic Accuracy Studies) [40], two employed PROBAST (Prediction Model Risk of Bias Assessment Tool) [41], and two utilized self-designed methods derived from well-recognized techniques [20, 24]. Regarding data synthesis, six conducted only descriptive analysis. Among the four methods that performed meta-analysis are: random-effects network meta-analysis [28], Bayesian meta-analysis [30], hierarchical summary receiver operating characteristic curve (ROC) [24], and linear mixed-effects meta-analysis [32]. The performance metrics extracted from their selected models were mostly area under the receiver operating characteristic curve (AUC) or accuracy.

Regarding the coverage and focus on CVD of these articles, only two of the selected publications discuss the exact task of interest [11, 32]. 16 investigate risk prediction associated with a broad definition of CVD outcomes and tasks. 4 concentrate on risk prediction for single CVD subtypes as part of primary prevention: 3 on heart failure (HF) [29, 30, 37] and 1 on stroke [25].

All the publications selected discuss the use of EHRs to some extent. 2 of the publications dedicate a section to thoroughly discussing the use of EHRs in CVD risk prediction [23, 33]. 15 publications discuss the integration of EHRs in ML-based CVD prediction within the text, usually highlighting the opportunity of integrating EHR with ML. 5 others mention EHRs and use EHR-based models as a reference but do not fully explore this topic.

Since the selected publications were primarily systematic reviews and reviews, the ML/DL methods considered typically encompassed a range of models. The specific models discussed were determined by the literature selected for review. 6 of these publications include discussions comparing conventional models with ML methods [17, 18, 29, 30, 32, 33].

### 3.3 Opportunities and Challenges Identified by the Selected Studies

The existing literature has been limited in its discussion of ML-based CVD risk prediction leveraging EHRs in the outpatient setting, particularly regarding the 10–15-year risk of CVD in undiagnosed patients—an area that holds potential for primary prevention strategies. This review addresses this gap by focusing exclusively on this specific domain and exploring the potential to replace existing conventional risk assessment tools. The opportunities and challenges, summarized from selected publications in the Table 3, relate to the nature of CVD, the characteristics of EHRs, and the complexities inherent in ML/DL models.

**Table 3.** **Opportunities and challenges in machine learning-based cardiovascular diseases prediction algorithm using electronic health record**.

Domain	Opportunity	Challenge
Cardiovascular disease	Early intervention and treatment	Complexity
	- Asymptomatic in early stages of CVD development.	- CVD is a complex, multifactorial disease with many interrelated risk factors that can be difficult to disentangle and quantify.
	- Patients benefit from early intervention.	- CVD patients are a highly heterogeneous group, with varying risk factors, clinical presentations, and responses to treatment.
	Personalized medicine	Data source
	- Identify individualized risk factors and profiles.	- CVD data are often incomplete, heterogeneous, and noisy.
	- Tailoring prevention and treatment strategies.	- Accessing and integrating data from multiple sources is challenging in practice.
	Cost-effective	Adaptability
	- Prioritize screening and intervention efforts.	- Prediction should be adaptable to different manifestations of CVD in different patients’ groups.
	- Allocate resources both efficiently and effectively.	- Predictive model should incorporate new risk factors.
	Improve understanding of underlying mechanisms of CVD	
	- Identify new risk factors or relationships between known risk factors and CVD outcomes.	
	- Help researcher develop new hypotheses and design more targeted studies.	
Electronic Health Records	Comprehensive and diverse datasets	Data quality and completeness
	- Rich individualized data sources that include background characteristics, medical history, laboratory results, prescribed therapies, and diagnoses.	- Data from different EHR sources can vary in quality and completeness.
	- Large sample sizes featuring diverse patient populations, enhancing generalizability.	- Unstructured and heterogenous nature of some EHR sources.
	- The provision of more complete records, reducing the proportion of missing data related to CVD events, risk factors, and comorbidities.	- Imbalance data for some CVD outcomes.
	Real-time longitudinal data	Interoperability and standardization
	- Data linkage techniques can generate longitudinal health data.	- Different data formats, codes, and controlled terminologies need mapping.
	- Timely identify patients at high risk of CVD events.	Data privacy and security
	EHR phenotyping	- Contain sensitive patient information.
	- Standardized platform for extracting, disseminating, and reusing clinical information.	- Anonymous data can still be traced back to individuals.
Machine/Deep learning	Improved accuracy	Inconsistent performance
	- ML may provide superior predictive capabilities for specific CVD events in subpopulations compared to conventional statistical models.	- The performance can vary significantly depending on the specific training dataset, features used, optimization of hyperparameters, and performance metrics employed.
	Flexibility	Overfitting
	- ML is capable for handling non-linear relationship between risk factors and outcomes, modelling complex and hidden patterns.	- Imbalanced CVD data.
	Personalization	- Model selection.
	- Can be tailored to specific individuals, enabling personalized predictions and therapy recommendation.	- Poor generalization on new data.
	Practical usability	Interpretability
	- Faster decision-making and response times with low computational cost.	- ‘Black box’ paradox.
	- Can handle large and complex datasets due to scalability.	- Code transparency and replication instructions.
	- The ability to learn from and adapt to new data sources.	- Apply the results in real-world healthcare scenarios.
		Ethical issue
		- Fairness, accountability, and transparency in healthcare.
		- Official guidelines and regulatory oversight.

#### 3.3.1 Cardiovascular Disease Outcomes

Given the complexity of CVD, a careful evaluation of previous publications and their specific CVD focus areas is crucial. Early intervention plays a critical role in the effective prevention and management of CVD, as patients are often asymptomatic during the early stages of the disease. In fact, early intervention and treatment have been shown to prevent up to 80% of heart disease and stroke events, underscoring the importance of identifying and managing risk factors as early as possible to prevent the development and progression of CVD [42]. All two studies focusing on HF emphasize that efforts should be shifted from stages C/D to stages A/B [29, 37].

Personalized cardiovascular medicine, enabled by ML and EHRs, represents a promising approach to tailoring therapy and treatment strategies. By identifying individualized risk factors and profiles, this approach ultimately leads to better patient outcomes [26, 34]. Moreover, it facilitates the prioritization of screening and intervention efforts at the population level, as well as the efficient allocation of public resources, making it a cost-effective option in clinical practice [27].

By leveraging large EHR datasets, ML algorithms can identify novel CVD risk factors and elucidate relationships between known risk factors and CVD outcomes. This leads to a more comprehensive understanding of underlying mechanisms and enables more targeted investigations. Furthermore, several reviews have discussed the possibility of predicting cardiometabolic diseases (including CKD and diabetes) collectively [43, 44], and some reviews have already reported studies on different cardiometabolic diseases, including CVD [45].

However, CVD is a complex and multifactorial disease characterized by many interrelated risk factors, which can be challenging to quantify. The considerable heterogeneity among individual patients with CVD further complicates the integration of data from multiple sources, especially when it involves sound, image, and genetic data. Some reviews have raised concerns that such complex models may become too overloaded [17, 20, 35].

Nonetheless, the application of ML algorithms in CVD research holds substantial potential for addressing these complexities and improving patient outcomes. It is crucial, however, to ensure that predictive models are adaptable to the various manifestations of CVD and that they incorporate new risk factors as they are identified. This approach will enable effective risk stratification and intervention. Continuous learning and improvement have been identified as challenges by some reviews, emphasizing the need for risk prediction models to be updated continually with developments in CVD research [33].

#### 3.3.2 Electronic Health Records Data Sources

The use of comprehensive and diverse individualized datasets is critical to developing accurate and effective ML based CVD risk prediction model. Most of the selected publications agree that EHR is recognized as a potential data source, which will be increasingly utilized for CVD prediction tasks in the future, potentially replacing traditional data collection methods [20, 23, 24, 25, 26, 27, 28, 31, 33, 35, 37]. EHRs should encompass diverse data sources, including background characteristics, medical history, laboratory results, prescribed therapies, and diagnoses. Additionally, they should feature large in-sample sizes and encompass patient populations that are generalizable across countries and settings [18, 23, 29, 37]. By utilizing such datasets, researchers can provide more complete records and reduce the proportion of missingness related to CVD events, risk factors, and co-morbidities [46, 47].

Real-time longitudinal data is also important as data linkage techniques of EHR can generate longitudinal health data that can be used to timely identify patients at high risk of CVD events [20, 48]. Furthermore, the use of EHR phenotyping provides a standardized platform for extracting, disseminating, and reusing clinical information. This can help improve the accuracy and consistency of data collection [49], which is essential for developing reliable ML models.

One major challenge in utilizing EHR data for CVD research is the issue of data quality and completeness [24]. Data from different EHR sources can vary in quality and completeness, and the unstructured and heterogeneous nature of some EHR sources can make it difficult to integrate data across different platforms [50]. While some studies have explored ML models leveraging EHR for CVD risk prediction [48, 50, 51], systematic evaluations remain limited, and existing research often lacks external validation or standardized methodologies. Moreover, the imbalance in data for some CVD outcomes has also raised concerns among researchers during the training of ML-based CVD models [22, 24].

Interoperability and standardization are also important challenges that must be addressed [52]. Different data formats, codes, and controlled terminologies need to be mapped and standardized to ensure that the data can be effectively integrated and utilized in ML models.

In addition, data privacy and security are significant concerns in using EHR data for CVD research [17, 26, 34, 37]. This is particularly true when the developed models require access to data for validation, a process that is unlikely to occur due to the current protection laws governing most EHR datasets [46]. EHR data contain sensitive patient information, and obtaining consent can cause selection bias [53]. Moreover, even anonymous data can potentially be traced back to individuals, creating potential risks to patient privacy [54]. To overcome these challenges, researchers must develop and implement appropriate data security and privacy protocols, as well as obtain necessary ethical and regulatory approvals [37].

#### 3.3.3 Machine/Deep Learning Technique

One major advantage highlighted in most of the selected publications is the potential for improved accuracy. These publications generally view ML-based models positively, noting their superior predictive performance in terms of both discrimination and calibration when compared to conventional approaches [11, 19, 20, 21, 25, 31, 32, 36, 37]. Specifically, ML algorithms are reported to offer enhanced predictive capabilities for CVD events in subpopulations, surpassing traditional statistical models. Moreover, numerous individual studies have demonstrated the superior performance of DL or neural network models over other ML models [11, 17, 19, 20, 22, 25, 28, 35]. Additionally, other ML techniques, such as SVM and ensemble methods—particularly boosting algorithms—have been frequently mentioned in systematic reviews as offering optimal performance [11, 19, 24, 25, 28].

ML demonstrates remarkable flexibility through its ability to manage non-linear relationships between risk factors, or covariates, and CVD outcomes. It effectively models complex and previously hidden interactions among various clinical and environmental variables, thereby accurately predicting desired CVD outcomes [19, 25, 29, 34, 37].

Furthermore, ML can be personalized for individuals by incorporating both individualized and community-level features [55], significantly enhancing the CVD prediction and therapy recommendation process [22].

ML models can efficiently extract data from large and complex datasets, providing timely responses at a relatively low computational cost compared to conventional approaches [22]. They are also capable of adapting to new data sources for continuous improvement and learning [33].

The use of ML in CVD research brings several advantages over conventional statistical models, but it also presents challenges that must be addressed. One notable challenge is the performance variability of ML models, which can be influenced by factors such as the demographic characteristics of the data source, the selection of features, the optimization of hyperparameters, and the choice of performance metrics [11, 21, 25]. This variability often leads to statistical heterogeneity in reported results, a problem that is extensively discussed in the literature.

Typically, studies assess the performance of different ML models by analysing each CVD subtype individually, subsequently choosing the best-performing model for that subtype. This method, while practical, introduces the risk of selection bias, as the chosen model might not be universally superior across all contexts. Furthermore, selected SRs have highlighted difficulties in identifying the optimal ML models from their evaluated development papers [20, 27]. These challenges arise from inconsistent performance comparisons, attributable to the fact that their included studies define different types of CVD [21, 22, 35]. Moreover, concerns arise regarding whether the performance differences between ML models and conventional statistical approaches are statistically significant [21].

Another challenge arises when an ML model is trained on a dataset without properly addressing the imbalance in CVD data. Its flexible nature may lead to overfitting the training dataset [20, 22], which, in turn, results in poor generalization to new data sources, especially for minority ethnic groups.

In addition, the interpretability of flexible ML models presents a concern, often referred to as the ‘black box’ paradox, which is undoubtedly a major challenge acknowledged by researchers. However, issues with the ‘black box’ extend beyond its inherent nature. A lack of interpretability can also stem from developers failing to disclose technical features. Selected studies have identified flaws in model development, including the absence of clear definitions and measurements for predictors [35], a lack of data-driven feature selection [19, 20], and insufficient details on hyperparameter tuning [24, 35]. This omission of technical details, coupled with a lack of replication instructions by the developers33, breaches the principle of code transparency [17, 25, 27, 35, 36, 37]. Consequently, it makes reproducing the models challenging and hinders their interpretability [17, 23, 31, 35]. Besides these technical reasons, there are also challenge before clinical application. Studies have report that several challenge, firstly, the different definition of CVD outcome may hard to be interpret in clinical settings [18, 35]. Also, selected studies also mention that the lack reporting of clinical features for model developer [22, 24, 35, 36].

Most ML-based CVD prediction models report their performance using the AUC, also known as the c-statistic, to demonstrate the models’ ability to discriminate [17, 20, 24, 25, 30, 31, 32, 35, 37]. But the clinical value of AUC value is hard to interpret. One review criticized that the healthcare impact of those ML models is overestimated and unrelated to patients’ clinical benefits [21]. While AUC quantifies a model’s discriminatory capacity, it provides an incomplete assessment of clinical utility [21, 24]. A robust predictive model should demonstrate strong calibration, reliability, and balanced trade-offs across multiple performance metrics [30, 32, 35]. Calibration, assessed through the Brier score, calibration curve, and calibration slope, reflects how well predicted probabilities correspond to actual outcomes. However, calibration metrics are often underreported, limiting comprehensive model evaluation [25, 36, 45]. Similarly, sensitivity, specificity, precision, recall, and F1-score capture different aspects of performance, yet selective reporting skews comparative assessments [25, 31, 45]. Given these limitations, integrating multiple metrics provides a more rigorous and clinically relevant assessment of ML-based CVD risk prediction models.

Ethical considerations are less frequently discussed in the selected publications, though they are crucial before the final application of ML in CVD prediction in real healthcare settings.

The first consideration is fairness. Health equality might be compromised by incorporating ML-based risk scores. It is noteworthy that most of the selected publications report that the ML models are predominantly developed by researchers in Western countries, focusing on populations of white ethnicity [17, 18, 21, 23, 25, 33, 34, 35, 37]. This situation could result in ‘indicate bias’, particularly disadvantaging patients in rural areas or those who are ethnic minorities. Additionally, these techniques could be inaccessible to patients with limited digital literacy [27].

Another consideration is accountability. Studies have observed a lack of consensus regarding the clinical effectiveness and safety of ML in practice. To date, none of the ML-based studies have conducted any clinical utility tests, and with the lack of clinical impact assessment for these risk prediction models, their usefulness in healthcare settings remains unknown.

Furthermore, the transparency of the ML algorithms themselves is uncertain; few clinicians are involved in the development of ML models, nor do they provide feedback to the developers. This issue has been reported by several selected studies [11, 26, 27, 34, 37].

These ethical issues highlight a significant gap in specific guidelines (e.g., NICE, AHA/ACC) for the development and implementation of ML in healthcare. Moreover, national regulatory oversight (e.g., Medicines and Healthcare Products Regulatory Agency (MHRA), Food and Drug Administration (FDA)) is essential to ensure standardized development, along with the clinical effectiveness and safety of ML. This requirement has been recognized by multiple selected studies [17, 18, 23, 24, 27, 35, 36, 37].

## 4. Discussion

A significant number of conventional CVD models utilizing EHR, or registry data have been developed, validated, and implemented in practice, with several high quality systematic reviews reporting on such models [56, 57, 58]. Numerous studies and researchers contend that expending extensive effort to develop new risk models is unnecessary at this stage [56, 59]. In contrast, systematic reviews focusing on ML-based models are scarce [32]. Furthermore, to our knowledge, no systematic or narrative review has specifically targeted ML-based CVD prediction models using EHR data for primary prevention.

The debate continues regarding whether ML-based approaches offer superior performance. At present, conventional models still play a significant role in clinical practice due to their simplicity and interpretability. The recently published updates of QRISK4 [9] (derived from UK EHR data) and the Predicting Risk of Cardiovascular Disease Events (PREVENT) equations [60] have demonstrated improved performance over previous conventional models, with the potential to enhance their clinical utility. This underscores the adequacy of conventional models for CVD primary prevention at present. However, given ML’s capacity for continuous refinement and long-term potential, a systematic evaluation of its role in risk prediction is warranted. Rather than viewing ML as an immediate replacement, delineating how it can complement and ultimately augment existing approaches is pivotal for advancing CVD risk prediction.

While some studies suggest that ML/DL outperforms conventional models, the need for appropriate independent external validation of any improvements is still not fully addressed [19, 20, 23, 29, 30, 35, 36, 37, 55]. This issue also applies to comparisons between different ML models. Thus, the development of ML-based models should be approached with focus on clinical utility, ensuring a responsible translation from research to real-world applications. The objective is not to supplant conventional models but to facilitate their gradual integration where ML demonstrably adds value. As ML advances, proactive measures—such as standardization, validation frameworks, and enhanced interpretability—are essential for its future adoption. An effective ML-based model should be both transparent and standardized, particularly when leveraging complex EHR data. Improved explainability will enable replication and iterative refinement, ensuring continuous advancement throughout its lifecycle.

### 4.1 Prospects of ML Based CVD Risk Prediction Model

The ‘last mile’ problem refers to the situation where the final step of operationalizing a concept into the real world proves to be the most complex and costly.

**Fig. 2.**
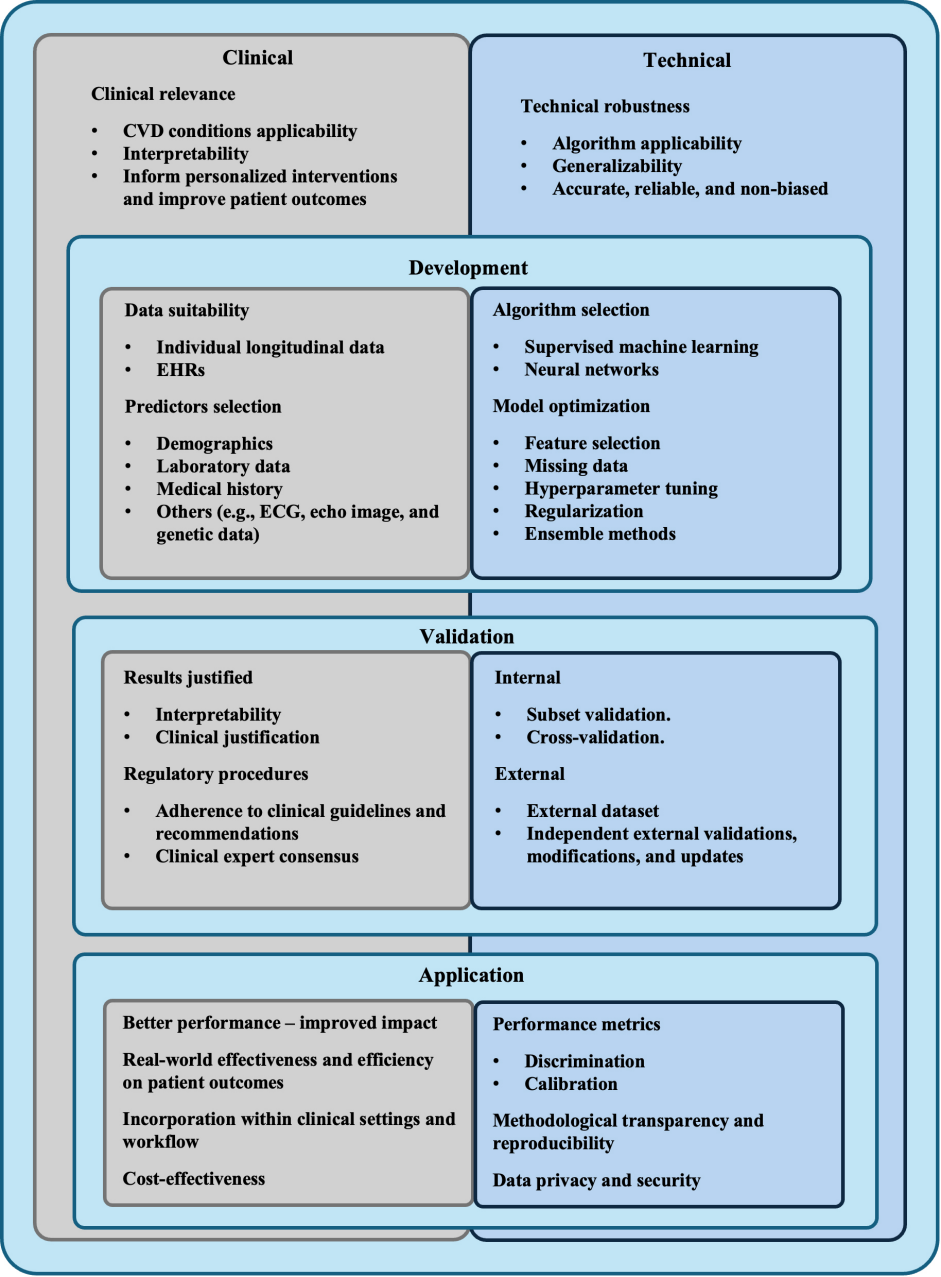
**An integrative framework for development, validation and application of machine learning based cardiovascular diseases risk prediction algorithm**.

Therefore, developing an integrative framework for the development, validation, and application of ML-based CVD risk prediction algorithms, as illustrated in Fig. 2, is crucial. Such framework should incorporate both clinical and technical perspectives, guiding researchers in creating practical and effective models that truly benefit clinicians and patients. While not exclusive to EHR-based ML models, use of EHR enhances the implementability of such frameworks.

#### 4.1.1 Clinical Relevance

Developing ML models for CVD requires a focus on clinical relevance and interpretability to ensure they meet the needs of patients and can be effectively used by clinicians for timely interventions. The integration of such models into clinical practice must address concerns around clinical workflow, model validity, and overall value to patient care [22, 24, 35, 36]. Utilizing EHR data for model development calls for transparent reporting of data use, including the phenotyping, mapping, and linkage of data items.

Predictor selection should consider demographics, laboratory data, medical history, and other relevant factors, with detailed reporting on measurement units and criteria. Ensuring transparency at this stage is key to the reproducibility and clinical utility of the models [17, 25, 27, 37].

Validation of these models should yield interpretable and justifiable results, in line with established clinical guidelines from organizations like the ACC/AHA, NICE, and ESC. Although specific reporting guidelines for healthcare ML models are in development, initiatives like TRIPOD-AI [61] are emerging to fill this gap.

Involving healthcare professionals and domain experts is critical for assessing a model’s clinical usability and ensuring its alignment with real-world practice [11, 26, 27, 34, 37]. Prior to clinical application, a model’s performance and impact must be evaluated against existing standards. Its integration into clinical workflows should be feasible, with consideration of healthcare providers’ and patients’ acceptance, as well as its cost-effectiveness analysis.

#### 4.1.2 Technical Robustness

Key considerations for ensuring the technical robustness of CVD risk prediction ML models include selecting an algorithm fit for the task, which should be effective across diverse patient demographics to avoid bias and ensure generalizability [11, 21]. Accurate, reliable, and unbiased performance is also essential, yet details on these factors are often inadequately reported [17, 23, 24, 30].

In development, the choice of ML model should balance performance with computational practicality [34]. Despite a large sample size negating the impact of algorithm choice on predictive accuracy, optimization techniques are vital for preventing overfitting and ensuring generalization.

The complexity of the models should be weighed against their ease of use and understanding. Developers need to document technical details, such as optimization techniques used, to ensure transparency and reproducibility [31, 35, 36, 45].

When implementing models in clinical settings, thorough evaluation using proper metrics, including probabilistic calibration and classification-based confusion matrix metrics, is necessary [30, 37]. Transparency in the model’s decision-making process is also critical, alongside compliance with data privacy and security measures. The lack of specific regulations for ML-based models calls for the development of comprehensive frameworks to maintain ethical standards in healthcare applications.

#### 4.1.3 Summary 

Developing, validating and applying ML models for CVD requires that both clinical and technical aspects are considered. Ensuring robustness and reliability enhances the trustworthiness for clinical use. Rigorous development and validation processes establish performance and effectiveness, instilling greater confidence in application. Identification of limitations and continuous evaluation lead to improvements and advancements in CVD prediction and management. Thus, frameworks are needed to support this process and yield more reliable, accurate, and impactful models, driving innovation and improving patient primary prevention of CVD.

### 4.2 The Definitive EHR Platform for ML Based CVD Risk Prediction Model

To advance ML-based CVD risk prediction, we propose a regulated, open-source EHR platform designed for standardized model development, evaluation, and validation. This initiative addresses key challenges in ML adoption by ensuring high-quality, representative multi-source data, promoting fair comparisons, enforcing regulatory oversight, and facilitating real-world clinical integration.

A fundamental limitation of current ML research is data heterogeneity and lack of representativeness, particularly concerning minority populations and underrepresented clinical cohorts. Existing open-source datasets are often insufficient in size, quality, or diversity, leading to biased models with limited generalizability. This platform would provide a large-scale, curated EHR dataset, encompassing structured and unstructured clinical data (demographics, medical history, lab results, prescriptions, and lifestyle factors), ensuring population representativeness and robust external validity.

A key feature is standardized benchmarking, addressing the lack of reproducibility and selective reporting in ML-based CVD risk prediction. All models would be evaluated using predefined metrics, including discrimination (AUC), calibration (Brier score, calibration curves), fairness audits (across demographic subgroups), and interpretability assessments. Developers must upload models, including all the technical features, the training strategy, and results, ensuring transparency, open peer review, and performance tracking, akin to version control systems. To enable fair comparisons, ML models would be benchmarked against conventional risk scores such as QRISK and Framingham, providing an objective reference standard.

Beyond research, this platform supports real-world clinical validation, bridging the gap between retrospective ML studies and clinical implementation. Top-performing models could undergo prospective validation or pilot implementations in hospital settings and be integrated into clinical decision-support systems, enabling systematic assessment of their impact on patient outcomes. Additionally, various machine learning models can be assessed by the trade-off between interpretability and performance in real-world clinical settings. By eliminating fragmented regulatory pathways, this initiative facilitates scalable, structured ML adoption, allowing policymakers to assess models through real-world performance and practical applicability rather than theoretical benchmarks, which are hard to replicate and interpret.

Ethical and regulatory compliance is a core component. The platform establishes a governance framework, mandating transparent reporting of methodologies, data usage, and compliance with regulatory standards. Unlike traditional models developed *in silos*, this initiative enforces strict privacy protections while enabling standardized fairness audits and bias mitigation strategies. By consolidating data access, it removes the need for multiple data-use agreements, streamlining research while ensuring accountability and regulatory oversight.

This platform also enables continuous improvement, allowing researchers to refine models based on prior results, incorporate advances in explainable AI, and explore privacy-preserving federated learning techniques. By providing a structured evaluation ecosystem, ML research can shift from ad-hoc comparisons to a standardized, iterative development pipeline, ensuring long-term adaptability and responsible innovation.

By centralizing model development, enforcing transparency, integrating regulatory oversight, and supporting clinical validation, this initiative establishes a structured pathway for ML integration into cardiovascular risk assessment, ensuring that future advancements are scientifically rigorous, clinically actionable, and equitably deployed.

### 4.3 Limitation

The limitations of this review include potential publication and reporting biases in the selected literature, as no formal quality assessment was conducted. The included studies may be inclined to report favourable results; however, this review qualitatively synthesizes the commonly identified opportunities and challenges across the selected studies rather than assessing biases within individual model reports, which helps contextualize potential reporting discrepancies. Another limitation is that the selected publications span a wide range of tasks and explore various CVD outcomes in diverse populations across different healthcare settings, using various data types. Only a few studies included in their reviews are directly relevant to the specific interests being addressed here. Despite this limitation, the review can still provide valuable insights, as most tasks in this field share similarities and common challenges. Additionally, some publications do not discuss EHRs comprehensively. Nevertheless, we have identified most of their attitudes toward EHRs. Considering that EHRs are used for more than this single task and are rapidly evolving in this field, we find that recent literature pays more attention to this aspect. Finally, the integrative framework we introduce has not been strictly examined and approved by any authority. However, we aim to facilitate its validation in the future to solidify the evidence base. At this point, we can only hope that it serves as inspiration for the future direction of researchers in this area.

## 5. Conclusions

ML-based CVD risk prediction models derived from EHRs offer immense potential but must overcome significant challenges to be clinically relevant and technically robust. Aligning with medical knowledge and clinical guidelines is crucial, as models need to be interpretable for trust and understanding. The points discussed in this review, including the latest techniques, data sources, model validation, and performance evaluation, should help guide the development of these models. Balancing computational cost, performance, and interpretability leads to the development of risk prediction algorithms that not only benefit patients but also advance scientific understanding.

Input from healthcare professionals and domain experts is invaluable in evaluating the models and identifying areas for improvement. Future research should be directed towards exploring the efficacy, usability, and impact of ML-based CVD prediction leveraging EHRs.

## Abbreviations

ACC/AHA, American College of Cardiology/American Heart Association; CKD, chronic kidney disease; CVD, cardiovascular disease; DL, deep learning; DM, diabetes mellitus; ED, emergency departments; EHRs, electronic health records; ESC, European Society of Cardiology; GP, general practitioner; HF, Heart failure; ICD, International Classification of Diseases; MI, myocardial infarction; ML, machine learning; NHS, the National Health Service; NICE, The National Institute for Health and Care Excellence; NIH, National Institutes of Health; NN, neural network; ROC/AUC, receiver operating characteristic curve/area under the ROC curve.

## Supplementary Material

Supplementary material associated with this article can be found, in the online version, at https://doi.org/10.31083/RCM37443.

## Appendix

See Appendix A.

**Search Strategy**

1. cardiovascular disease$.mp. or exp Cardiovascular Diseases/

2. exp Machine Learning/

3. (artificial intelligence or AI or machine learning or deep learning or neural network$).ti,ab.

4. exp Natural Language Processing/

5. (2 or 3) not 4

6. exp Risk Assessment/ or exp Risk Factors/

7. (predict$ or risk or score$ or model$ or algorithm$).ti.

8. 6 or 7

9. exp Electronic Health Records/

10. (electronic health record$ or electronic medical record$).mp.

11. 9 or 10

12. exp Review/ or exp Systematic Review/

13. 1 and 5 and 8 and 11 and 12
